# Adversity profiles of children receiving care and support from social services: A latent‐class analysis of school‐aged children in Wales

**DOI:** 10.1111/cch.13097

**Published:** 2023-01-31

**Authors:** Rebecca Anthony, Jonathan Scourfield, Graham Moore, Shantini Paranjothy, Annette Evans, Sinead Brophy, Rhian Daniel, Sara Long

**Affiliations:** ^1^ Centre for the Development, Evaluation, Complexity and Implementation in Public Health Improvement (DECIPHer), School of Social Sciences Cardiff University Cardiff UK; ^2^ Wolfson Centre for Young People's Mental Health Cardiff University Cardiff UK; ^3^ Children's Social Care Research and Development Centre (CASCADE), School of Social Sciences Cardiff University Cardiff UK; ^4^ Division of Population Medicine, School of Medicine Cardiff University Cardiff UK; ^5^ Centre for Health Data Science University of Aberdeen Aberdeen UK; ^6^ Health Data Research UK Swansea University Medical School, Swansea University Swansea UK

**Keywords:** adverse childhood experiences, care, child welfare, latent class analysis, social services

## Abstract

**Background:**

Children receive care and support from social services due to the risk of harm or impeded development or because of disability. This study aimed to identify typologies of adversity experienced by children receiving care and support from social services and to explore how typologies differ by sociodemographic characteristics.

**Methods:**

This is a cross‐sectional study of ‘Children Receiving Care and Support’ (*N* = 12 792) during 2017/2018 in Wales, UK. We sought to (1) examine the prevalence of household adversities experienced by children in receipt of care and support from social services; (2) identify typologies of household adversities; and (3) explore how typologies of household adversities differ by family characteristics (demographics, measures of social disadvantage, perinatal and care factors).

**Results:**

We found evidence for multiple risk factor constellations. The four‐class solution suggested four distinct classes of adversities: child disability (50.0%), low adversities (20.3%), family poor health (6.7%) and multiple risks (23.0%). Children in the ‘multiple risk’ class were significantly more likely to be younger, more deprived and ‘looked after’ by the local authority compared with those in the ‘low adversities’ class.

**Conclusions:**

Given the presence of different constellations of household adversities, policies and interventions that address multiple risk factors simultaneously may be more effective and have longer‐lasting benefits.

**Key messages**

- Family circumstances are complex and go beyond simply the co‐occurrence of domestic violence, substance abuse and parent with mental health problems.
- The presence of a group of children classified as ‘low adversity’ may suggest the system is over‐intervening.
- Half the children in our sample experienced deprivation, and this was more concentrated for children experiencing multiple adversities.
- Preventing the need for social services interventions through upstream actions to address common causes of underlying risk factors, such as child poverty, is needed.
- Interventions that match appropriate care and support to the specific constellation of family‐level factors in unison may bolster their effectiveness and have more substantial and long‐lasting benefits.

## 1 INTRODUCTION

Adverse childhood experiences (ACEs) represent early and sometimes chronic stressors, which can interfere with the development of healthy neural, immune and hormonal systems, as well as problematic psychological coping strategies for a review see (Sheffler et al., 2020). Numerous studies show a strong association between ‘adverse childhood experiences’ and poorer physical, social, mental health and educational outcomes during childhood (Evans et al., 2019; Felitti et al., 1998; Lowthian et al., 2021; Oh et al., 2018; Paranjothy et al., 2018) and later life (Hughes et al., 2017; Hughes et al., 2021; Nelson et al., 2020). Although the direction and nature of causality remain debated, studies reveal amplified rates of adversities for children who have experienced care (Lester et al., 2020; Turney & Wildeman, 2017) or have been adopted from care (Anthony et al., 2019). Recent research showed that many children who receive care and support from social services have complex histories of exposure to adversity (Conners‐Burrow et al., 2013), and requiring care and support even briefly is associated with substantially poorer educational outcomes (Berridge et al., 2020).

In Wales, where this study is based, for the year 2020, 2.6% of children were in receipt of care and support from social services (Welsh Government, 2021), with similar rates (3.2%) classified as ‘in need’ in England (UK Government, 2021). The primary reason for children receiving care and support from social services is the risk of, or experience of, abuse or neglect or because of family dysfunction. In line with the majority of countries, social services in the United Kingdom have the legal power to remove children from their parental home if there is a significant risk of harm that cannot be mitigated by providing support. Of the 16 580 children receiving care and support in Wales in March 2020, 6935 (41.8%) were ‘looked after’ by local authorities, and 2310 (13.9%) were on the child protection register (CPR), that is, identified as being at significant risk of harm.

In the last two decades, there has been an exponential increase in studies investigating adversity from a cumulative risk approach, as well as examining the constellation of adversities (Barboza, 2018). Some constellations (such as poverty and parental mental illness) have been shown to be associated with poorer outcomes (Lanier et al., 2018). Whilst a number of studies have investigated the comorbidity of adversities experienced by children in out‐of‐home care (Anthony et al., 2020; Baldwin et al., 2019; Keller et al., 2007; Pears et al., 2008; Petrenko et al., 2012; Warmingham et al., 2019), the wider group of children who receive care and support from social services for a range of reasons have received very little attention. Examining household adversities experienced by the wider group of children in receipt of care and support from social services can help with planning services based on families' needs and inform tailoring of existing interventions and future intervention development to help lessen the impact of early and prolonged stressful experiences.

The present study aimed to (1) examine the prevalence of household adversities experienced by children in receipt of care and support from social services; (2) explore typologies of adversities; and (3) examine how typologies differ by family characteristics (including demographics, measures of social disadvantage, perinatal and care factors).

## 2 METHOD

### 2.1 Design and data sources

Aims 1 and 2 use secondary data from the ‘Children Receiving Care and Support’ Census (CRCS), an administrative dataset of children (under the age of 18) in Wales, UK, identified as requiring care and support. This includes all children with a ‘care and support plan’ in place for 3 months or more on the 31 March each year. The CRCS includes all children receiving support financed from children's social services budgets (see Figure S1). We accessed CRCS datasets through the Secure Anonymised Information Linkage (SAIL) databank at Swansea University, UK (Ford et al., 2009). For aim 3, which explored how typologies of adversities differ by family characteristics, the CRCS census was anonymously linked to the Wales Electronic Cohort for Children (WECC) (Hyatt et al., 2011). The WECC has records for approximately one million children born between 1990 and 2012 for a child or mother resident in Wales with information held in the Wales Demographic Service Dataset (WDSD) (a Wales‐wide administrative register for all individuals with a general practitioner [GP]) (Paranjothy et al., 2018). WECC is derived by record‐linking deidentified routinely collected health and social data sets using a unique Anonymised Linking Field (ALF) for each individual (Lyons et al., 2009). This study received ethical approval from the School of Social Sciences Research Ethics Committee in Cardiff University, and the analysis of linked data from the Wales Electronic Cohort for Children was approved by an independent Information Governance Review Panel.

### 2.2 Variables

To examine the prevalence and typologies of adversities experienced by our population of interest, we included seven binary variables recorded in the CRCS census to closely match the original ‘Adverse Childhood Experiences’ (Felitti et al., 1998). These were as follows: indicators of the child experiencing or being at risk of experiencing abuse (on the CPR register); domestic abuse; parental mental ill health (any mental health problems diagnosed by a medical practitioner; self‐reported problems; and parents receiving services from the Community Mental Health Team); and parental substance abuse. Additional adversities included: the child having a recorded disability, as a recent report suggested that the ‘Adverse Childhood Experiences’ framework ignores other sources of adversity, including disability (Welsh Government, 2021); and parental learning difficulties associated with lower child well‐being (Neil et al., 2020). See Table 1 for adversity definitions.

**TABLE 1:** Key variables as defined in the children receiving care and support census.

Variable type/name	Description
Potential adversities	
Asylum seeker	A true/false field to indicate whether or not a child has been an asylum‐seeking child, that is, an application has been made for protection on the basis of the Refugee Convention or Article 3 of the European Convention on Human Rights and who is awaiting a decision on that application at any point during the period 1 January to 31 March within the year of the return.
Child disability	A True/False field indicating whether the child was disabled on 31 March 20XX. For the purposes of this item, the definition of disabled follows that of Section 6 of the Equality Act 2010, which states that: ‘A person (P) has a disability if (a) P has a physical or mental impairment and (b) the impairment has a substantial and long‐term adverse effect on P's ability to carry out normal day‐to‐day activities’. Disability type includes: autistic spectrum disorder; continence; ability to lift, carry or otherwise move everyday objects; manual dexterity; memory; mobility; perception of the risk of physical danger; physical co‐ordination; speech, hearing and eyesight.
Domestic abuse	A True/False field indicating whether domestic abuse, was present on 31 March 20XX. Counted as true if one or more of the child's parents or carers has domestic abuse problems. Domestic abuse is physical, sexual, psychological or financial intimidation, violence or threats of violence that take place within an intimate or family‐type relationship and that form a pattern of coercive and controlling behaviour. This can include forced marriage and so‐called ‘honour crimes’.
Parental learning disability	A True/False field indicating whether the parental learning disability was present on 31 March 20XX. Counted as true if one or more of the child's parents or carers has domestic abuse problems. Counted as true if one or more of the parents or carers has an impairment of intellectual function that significantly affects their development and leads to difficulties in understanding and using information, learning new skills and managing to live independently. 1 = True; 0 = False.
Parental mental ill health	A True/False field indicating whether the parental mental ill health was present on 31 March 20XX. Counted as true if one or more of the parents or carers has a mental health problem. Include mental health problems diagnosed by a medical practitioner; self‐reported problems; and parents receiving services from the Community Mental Health Team. Include depression; self‐harming; and eating disorders. Exclude substance misuse, and Autistic Spectrum disorders and other learning disabilities.
Parental physical ill health	A True/False field indicating whether the parental physical ill health were present on 31 March 20XX. Counted as true if one or more of the child's parents or carers has physical health problems that impair their ability to care for the child.
Parental substance/alcohol misuse	A True/False field indicating whether the substance/alcohol misuse were present on 31 March 20XX. Counted as true if one or more of the parents or carers has a substance misuse problem, that is, intoxication by – or regular excessive consumption of and/or dependence on – psychoactive substances, leading to social, psychological, physical or legal problems. It includes problematic use of both legal and illegal drugs (including alcohol when used in combination with other substances). In this guidance document, the term ‘drug’ is used to refer to any psychotropic substance, including illegal drugs, illicit use of prescription drugs and volatile substances.

Family characteristic variables from the WECC include child age at the start of the census period, gender, congenital anomalies, child entitlement to free school meals during key stage one and maternal age. See Table 2 for information.

**TABLE 2:** Descriptive statistics.

Sociodemographic variables (*N* = 9960)		
Sex	*N*	%
Females	3648	36.6%
Males	4667	46.9%
Missing data	1645	16.5%
Age (range 5 to 18)	*M* = 11.54	SD = 0.033
Age category (developmental period)		
Early childhood (5 to 8)	2670	26.8%
Middle childhood (9 to 12)	3519	35.3%
Adolescence (13 to 18)	3771	37.9%
Missing	8	0.1%
Congenital anomalies		
None	8770	88.1%
Major	1037	10.4%
Minor	153	1.5%
Maternal age at birth		
<18	505	6.1%
18+	7790	78.2%
Missing data	1665	16.7%
Free school meal eligibility during KS1		
No	4339	43.6%
Yes	4872	48.9%
Missing data	749	7.5%
Childhood adversities (during census period) *N* = 12 792		
Disability	2623	29.0%
Seeking asylum	11	0.1%
Parental substance misuse	2362	26.2%
Parental learning disabilities	558	6.2%
Parental mental ill health	2780	30.8%
Parental physical ill health	1101	12.2%
Domestic abuse	2091	23.2%
Child protection register (CPR)	1197	13.3%
‘Looked after’ by local authority	3382	37.4%

### 2.3 Study sample

To examine the prevalence and typologies of adversity, all children included in the most recent (2017/18) CRCS census available within the SAIL databank were included (*N* = 12 792). Our sample includes children who receive ‘short breaks’, that is, the provision of day, evening, overnight and weekend activities for the child or young person. This group of children is often excluded from studies of children ‘looked after’; however, as this study is interested in the wider category of children receiving any care and support, we took the decision not to exclude them. Aim 3 (to explore how typologies differ by family characteristics), which linked the CRCS dataset to WECC, included 9960 participants, as 22.1% (*n* = 2832) were not able to be matched with an ALF. See Figure S2 for sample details.

### 2.4 Statistical analysis

Stata version 16.0 was used to conduct descriptive statistics and perform latent class analysis using gsem (StataCorp, 2019). A three‐step approach was used to conduct the analysis. First, we tested the fit of two to five latent classes by comparing fit indices (Akaike information criterion [AIC], Bayesian information criterion [BIC] and entropy values across number of classes). Lower AIC and BIC values indicate a better fit, whereas an entropy value closer to 1 indicates a clearer delineation of classes. Second, once the optimal solution had been identified, participants were classified into latent classes according to the maximum posterior probability. Third, multinomial logistic regression predicted membership in latent classes relative to the ‘lower risk of measured adversities’ class (reference group) based on socio‐demographic, perinatal and care variables.

## 3 RESULTS

### 3.1 Aim 1: Prevalence of adversities

Adversities experienced by children in the CRCS are reported in Table 1. Within this sample, 13.3% (*n* = 1197) were on the CPR, and 37.4% (*n* = 3382) were currently ‘looked after’ by the local authority. Over a quarter (29.0%, *n* = 2623) were recorded as being disabled. In terms of household adversities, 26.2% (*n* = 2362) lived with a parent who abused substances, 30.8% (*n* = 2780) lived with a parent with mental health problems, 23.2% (*n* = 2091) lived in a home where there was domestic abuse, 12.2% (*n* = 1101) lived with a parent with physical health problems and 6.2% (*n* = 558) had parents with a learning disability. With regard to abuse and neglect, 13.3% (*n* = 1197) were on the Child Protection Register. A small number of children were recorded as seeking asylum (0.1%, *n* = 11); this variable was excluded from the analysis as numbers were too small.

### 3.2 Aim 2: Typologies of adversity

The four‐class solution had low AIC and BIC values, and the highest entropy levels (see Table 3); based on these results, we chose the model with four classes. The four‐class solution suggested four distinct classes of adversities, which we named: child disability (50.0%), low adversity (20.3%), family poor health (6.7%) and multiple adversities (23.0%). See Figure S1 for item response probabilities and Table 4 for item response probabilities and the cumulative number of adversities across class membership.

**TABLE 3:** Fit indices for latent class analysis of current potential life stressors.

	AIC	BIC	Entropy value
1‐class	85 235.97	85 288.16	‐
2‐class	79 680.88	79 792.73	0.914
3‐class	79 007.33	79 178.83	0.898
4‐class	78 558.76	78 789.91	0.963
5‐class	60 460.31	60 734.15	0.896

Abbreviations: AIC, Akaike information criterion; BIC, Bayesian information criterion.

**TABLE 4:** Item response probabilities, cumulative number of adversities and class membership proportions for the four‐class solution.

	Class 1 ‘child disability’	Class 2 ‘low adversities’	Class 3 ‘family poor health’	Class 4 ‘multiple adversities’
Child disability	**0.57**	0.02	**0.37**	0.06
Parental substance misuse	0.02	0.18	0.22	**0.82**
Parental learning disabilities	0.04	0.01	0.23	0.07
Parental mental ill health	0.08	0.18	**0.78**	**0.68**
Parental physical ill health	0.07	0.03	**0.50**	0.15
Parental domestic abuse	0.02	0.20	0.21	**0.70**
Child protection register	0.01	0.25	0.12	0.23
Cumulative number of adversities: Mean (SD), range	0.63 (0.68) range 0 to 3	1.16 (0.37) range 1 to 3	2.96 (0.76) range 2 to 7	2.86 (0.87) range 2 to 6
Class proportions	50.0% (*n* = 6402)	20.3% (*n* = 2592)	6.7% (*n* = 858)	23.0% (*n* = 2940)

*Note*: Item response probabilities over 0.3 have been bolded to illustrate the adversities most pertinent to each class.

#### 3.2.1 Class 1: Child disability

This class is made up of half the sample (50.0%, *n* = 6402). It was characterized by the highest probabilities of the child experiencing disability and low probability of any other measured adversities. Most of the disabled children were assigned to this class (*n* = 2454, 38.33%); 5.3% (*n* = 339) lived with a parent with physical health issues. This class experienced the lowest level of cumulative adversities, *M* = 0.63 (SD = 0.68), ranging from experiencing zero to three.

#### 3.2.2 Class 2: Low adversity

This class formed 20.3% (*n* = 2592) of the sample and was made up of children with low probabilities of exposure to adversities compared with other classes. The most prominent adversities were substance misuse; nearly one in four of the children in this class (18.36%, *n* = 476) lived with a parent with a substance misuse problem, 22.38% (*n* = 580) lived with a parent with mental health issues and 16.28% (*n* = 422) lived with domestic abuse. This class has the highest proportion of children on the CPR register (25.93%; *n* = 672). On average, this class experienced one adversity (*M* = 1.16, SD = 0.37, Range 1 to 3).

#### 3.2.3 Class 3: Family poor health

This smallest class, which formed just 6.7% (*n* = 858) of the sample, were distinguished by child and parent poor health. Most of the children (73.66%, *n* = 632) experienced a parent with mental health problems. Over half the sample experienced a parent with physical health problems (56.18%, *n* = 482), and 41.14% (*n* = 353) were disabled themselves. Around a third (30.54%, *n* = 262) experienced a parent with a learning disability, 13.05% (*n* = 112) experienced parental substance misuse, 19% (*n* = 163) experienced domestic abuse and 8.51% (*n* = 73) were on the CPR. This class experienced the highest level of cumulative adversities, *M* = 2.96 (SD = 0.76), ranging from two through to seven. In contrast to class 1, ‘child disability’, which had a high proportion of disabled children but a low risk of other factors, this class is characterized by a high proportion of child disability alongside other adversities mostly related to parental health.

#### 3.2.4 Class 4: Multiple adversities

This class formed 23.0% (*n* = 2940) of the sample. The multiple adversities class was characterized of children with high probabilities of living with parents who misused substances, had mental health problems and experienced domestic abuse. Within this group, 64.70% (*n* = 1902) lived with a parent experiencing substance misuse problems, over half (54.05%, *n* = 1589) lived with a parent with mental health issues, and 53.98% (*n* = 1587) experienced domestic abuse. Some (14.01%, *n* = 412) experienced a parent with a physical health condition, and 4.97% (*n* = 146) had a parent with a learning disability. This class had the second highest proportion of children on the child protection register (15.85%, *n* = 466). A small proportion of children (3.81%, *n* = 112) were disabled. This class also experienced high levels of cumulative adversities, *M* = 2.86 (SD = 0.87), ranging from experiencing two adversities through to six.

### 3.3 Aim 3: How typologies of adversities differ by family characteristics

Table 2 shows the demographic details of children included in this sample. The mean age was 11.54 years (SD 0.33). Table S1 shows the sociodemographic, perinatal and care descriptives by class membership. Table 5 shows the relative risk ratios (and 95% confidence intervals) from the multinomial logistic regression models.

**TABLE 5:** Multinomial logistic regression results of sociodemographic, perinatal and care predictors of latent class membership (*N* = 7784).

Family characteristics	Group 1: Child disability			Group 3: Family poor health			Group 4: ‘Multiple adversities’		
	RRR (SE)	95% CI	*p*	RRR (SE)	95% CI	*p*	RRR (SE)	95% CI	*p*
Child age									
Middle childhood	**1.38 (0.10)**	**1.20–1.60**	**0.000**	**1.58 (0.20)**	**1.23–2.03**	**0.000**	1.02 (0.09)	0.87–1.21	0.780
Adolescence	**1.84 (0.14)**	**1.58–2.15**	**0.000**	**1.90 (0.35)**	**1.46–2.46**	**0.000**	**0.88 (0.08)**	0.73–1.05	0.149
Gender – male	**1.39 (0.08)**	**1.23–1.57**	**0.000**	**1.38 (0.14)**	**1.13–1.69**	**0.002**	1.07 (0.08)	0.93–1.24	0.321
Free school meals	**0.52 (0.03)**	**0.46–0.59**	**0.000**	1.08 (0.11)	0.88–1.33	0.467	**1.22 (0.09)**	**1.06–1.42**	**0.007**
Maternal age (18+)	**1.47 (0.18)**	**1.15–1.87**	**0.002**	**2.01 (0.48)**	**1.25–3.22**	**0.004**	1.01 (0.14)	0.77–1.32	0.951
Congenital anomalies									
Major	**5.49 (0.83)**	**4.08–7.39**	**0.000**	**5.53 (1.06)**	**3.80–8.05**	**0.000**	1.38 (0.26)	0.94–2.00	0.097
Minor	**2.77 (0.84)**	**1.53–4.50**	**0.001**	1.78 (0.85)	0.70–4.52	0.222	1.20 (0.45)	0.58–2.49	0.625
Looked after child	1.04 (0.17)	0.72–1.39	0.539	**1.82 (0.19)**	**0.03–0.09**	**0.000**	**2.18 (0.16)**	**1.88–2.52**	**0.000**

*Note*: Ref class: Class 2 (lower adversity group).

Children in class 1 ‘child disability’ were significantly more likely to be older, a boy, have major and minor congenital anomalies and have a mother aged over 18 at birth, compared with class 2, the ‘low adversity’ reference group. This class was also less likely to receive free school meals and less likely to be ‘looked after’ by the local authority. Children in class 3 ‘family poor health’ were significantly more likely to be older children, a boy, to have a mother aged over 18 at birth, and to have major congenital anomalies compared with the low adversity reference class. They were also more likely to be ‘looked after’ by the local authority. Children in group 4, the ‘multiple adversities’ class, were younger, more likely to experience deprivation (i.e. receive free school meals) and be ‘looked after’ by the local authority compared with the ‘lower adversity’ reference group.

## 4 DISCUSSION

This study provides an insight into the social conditions experienced by families in receipt of social care and support in Wales. A recent review (Skinner et al., 2021) and research study (Hood et al., 2021) have questioned the evidence base for the association between child maltreatment and co‐occurring domestic abuse, parental substance misuse and mental health problems. The (Hood et al., 2021) study created demand typologies in social care using latent class analysis to explore child episodes where a social services assessment was undertaken—that is, a wider group than children receiving care and support in six English local authorities (Hood et al., 2021). They found evidence for seven classes, including three single factor classes and four classes made up of combinations of stressors, including child maltreatment, child/parent disability, mental health, domestic violence and substance abuse. Our study supports these findings that family circumstances are complex and go beyond simply the co‐occurrence of domestic violence, substance abuse and parent with mental health and international studies using latent class analysis to examine risk profiles (Browne et al., 2018). However, the high probability of coexistence of these three adversities in one of our classes, which comprised 23% of the sample, does suggest the importance of this scenario for a minority of children who receive care and support in Wales—a minority who are more likely to be looked after by their local authority. At the very least, this finding suggests that social care practice continues to acknowledge this kind of family situation.

The presence of a ‘family poor health’ class reflects previous research, which showed that in addition to elevated exposure to early adversity, children involved with child welfare were also more likely to experience complex health challenges, including physical, developmental and mental health problems (Rienks et al., 2017; Stein et al., 2013). Additionally, given that our study included children who receive ‘short breaks’, that is, the provision of day, evening, overnight and weekend activities, and we included child disability as an adversity variable, it is not surprising that there was a large class of children who were disabled alongside very low probabilities of experiencing any other adversities. It is important to note the size of this group and plan the resources to support these families. The presence of a low adversity class is interesting and perhaps contrary to expectations. One possible interpretation is that the system is over‐intervening. Rates of children looked after have been rising since the early 1990s (Thomas, 2018), and the cumulative incidence of referral to children's services is extremely high. Jay et al.'s (in press) research using administrative data found that 25% of all children in England were classed as ‘in need’ between birth and age 16, with 43% referred to social care over the same period. It is, however, also possible that our dataset does not capture a wide enough array of risk factors to fully understand their experiences and the reasons for receiving care and support. Future research should explore this aspect further to understand why these families are receiving care and support and the type of support received.

Extensive research has documented the relationship between deprivation and social services involvement; children living in low‐income families and deprived neighbourhoods are disproportionately represented within the care system (Elliott, 2020; Rebbe et al., 2017; Turney & Wildeman, 2017). The results from our study suggest that deprivation is relevant not just to child protection cases but to the wider category of children who need care and support from social services. Half of children in our sample were eligible for free school meals (compared with 17% in the Welsh population; Welsh Government, 2018), and this eligibility was especially concentrated in the family poor health and multiple exposures classes. These findings align with previous studies, which found that poverty was strongly related to both individual adversities and specific clustering of adversities (Lacey et al., 2022). These findings also speak of the importance of joining up children's and adults' social services so that support for parents with (e.g.) mental health problems or substance misuse is better integrated with an intervention that is focused on child welfare, and the experience and expertise of practitioners with these different specialisms are pooled. Such integration has been attempted in Wales in Integrated Family Support Services, which tackle parental substance misuse where children are at high risk of becoming looked after but not elsewhere in the system. Better joint working of social and health services is also very much needed to respond to complex child and family needs (Afzelius et al., 2018; Van Dongen et al., 2018). There are institutional barriers, but if these could be overcome, the benefits could be considerable (Children's commissioner for Wales, 2020).

### 4.1 Strengths and limitations

The use of routine data has excellent opportunities, including large sample sizes and the ability to consider aspects such as individual‐level deprivation. Whilst latent class analysis has been highlighted as a method for social workers to understand in order to best study prevention (Lippold et al., 2017), the typologies created are limited by the available data (Petersen et al., 2019), which only included household adversities recorded within the CRCS census and will inevitably miss information about a child's life (Farmer & Dance, 2016). Studies have found that including wider ACEs at the community level, such as poverty, neighbourhood cohesion and experiencing discrimination, can add to our understanding of the impact of chronic stressors (Cronholm et al., 2015; Lacey et al., 2022). In addition, a three‐step rather than a one‐step approach was used for the analysis due to software restrictions, which can lead to biased estimates (Bolck et al., 2004).

Within the CRCS census, the eligibility criterion that children must have a care and support plan on the census date of 31 March (of any given year) that must have been in place for the previous 3 months means that the total number of children included in the CRCS census is less than the actual number of children receiving care and support. Furthermore, the data available in SAIL contain only those children with a Unique Pupil Number (UPN) to allow anonymous matching of children with the National Pupil Database (NPD). Therefore, data are missing for children who have not yet entered school. This is important as children on the child protection register were generally younger than other children receiving care and support, with 37% aged under 5 (Welsh Government, 2019); thus, some variables may be underestimated by the absence of the youngest children.

### 4.2 Conclusion

Given the association between risk of experiencing clusters of adversity and deprivation, we suggest that preventing the need for social services interventions in the first place through upstream actions to address common causes of underlying risk factors, such as child poverty, is needed. Alongside this, interventions that match appropriate care and support to the specific constellation of family‐level factors in unison may bolster their effectiveness and have more substantial and long‐lasting benefits (Fals‐Stewart et al., 2004).

## CONFLICT OF INTEREST STATEMENT

We have no conflict of interest to disclose.

## ETHICS STATEMENT

This study received ethical approval from the School of Social Sciences Research Ethics Committee in Cardiff University.

## Supporting information


**Table S1.** Sociodemographic, perinatal and care descriptives, by class membership
**Figure S1.** Venn diagram to represent children included in the ‘Children in Receipt of Care and Support’ census dataset
**Figure S2.** Study sample

## Data Availability

All data are available through the SAIL gateway (www.saildatabank.com).
