# Supplementary material for: Adversity profiles of children receiving care and support from social services: A latent‐class analysis of school‐aged children in Wales
Source: Child Care Health Dev. 2023 Jan 31;49(5):889–97. doi: 10.1111/cch.13097 (PMC10946723; doi:10.1111/cch.13097)

**Appendices**

**Online table 1**

*Sociodemographic, perinatal and care descriptives, by class membership*

|  | **Class 1 ‘Child disability’** | **Class 2 ‘Low adversity’** | **Class 3 ‘Family poor health’** | **Class 4 ‘Multiple adversities’** |
| --- | --- | --- | --- | --- |
| Gender |  |  |  |  |
| Female | 1,772 (40.6%) | 774 (48.9%) | 242 (42.2%) | 860 (48.0%) |
| Male | 2,594 (59.4%) | 809 (51.1%) | 332 (57.8%) | 932 (52.0%) |
| Age category |  |  |  |  |
| Early childhood | 1,232 (23.7%) | 603 (31.8%) | 149 (21.2%) | 686 (31.6%) |
| Middle childhood | 1,796 (34.6%) | 676 (35.7%) | 263 (37.5%) | 784 (36.1%) |
| Adolescence | 2,162 (41.7%) | 616 (32.5%) | 290 (41.3%) | 703 (32.4%) |
| Free school meals |  |  |  |  |
| No | 2,709 (56.4%) | 683 (38.9%) | 256 (40.0%) | 691 (34.4%) |
| Yes | 2,093 (43.6%) | 1,075 (61.2%) | 384 (60.0%) | 1,320 (65.6%) |
| Maternal age |  |  |  |  |
| Under 18 | 225 (5.2%) | 118 (7.5%) | 25 (4.4%) | 137 (7.7%) |
| 18+ | 4,129 (94.8%) | 1,461 (92.5%) | 548 (95.6%) | 1,652 (92.3%) |
| Birth abnormalities |  |  |  |  |
| None | 4,282 (82.5%) | 1,822 (96.2%) | 591 (84.2%) | 2,075 (95.5%) |
| Minor | 105 (2.0%) | 14 (0.7%) | 13 (1.9%) | 21 (1.0%) |
| Major | 803 (15.5%) | 59 (3.1%) | 98 (14.0%) | 77 (3.5%) |
| Child 'looked after' |  |  |  |  |
| No | 3,447 (66.4%) | 1,254 (66.2%) | 379 (54.0%) | 1,052 (48.4%) |
| Yes | 1,743 (33.6%) | 641 (33.8%) | 323 (46.0%) | 1,121 (51.6%) |

**Online Figure 1.**

*Venn diagram to represent children included in the ‘Children in Receipt of Care and Support’ census dataset*

**
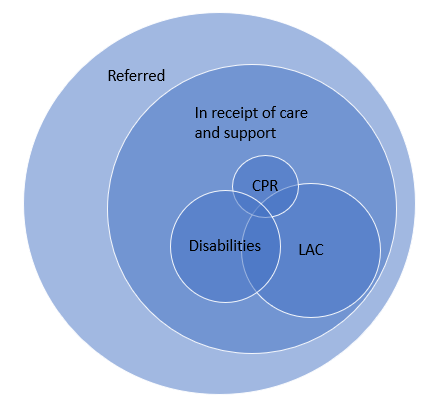
**

**Online Figure 2.**

*Study sample*


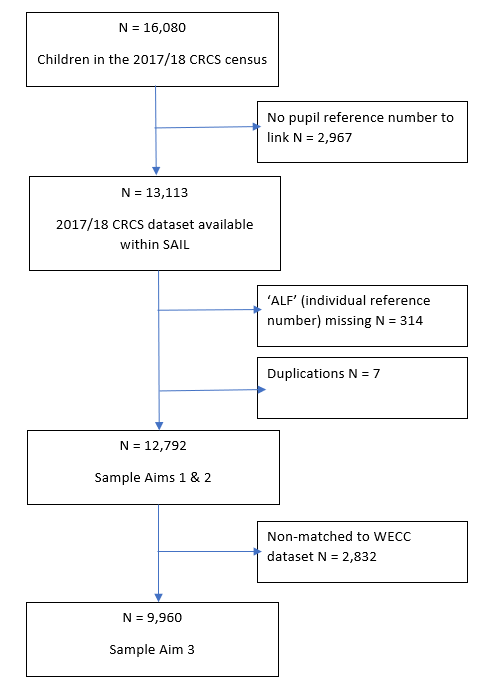

Supplement: Supplementary file 1 — Table S1. Sociodemographic, perinatal and care descriptives, by class membership Figure S1. Venn diagram to represent children included in the ‘Children in Receipt of Care and Support’ census dataset Figure S2. Study sample [file CCH-49-889-s001.docx]
